# Cytokine and immunoglobulin profiles of Arbor Acres broiler chickens at different stages of physiological development

**DOI:** 10.14202/vetworld.2024.988-993

**Published:** 2024-05-04

**Authors:** Svyatoslav Lebedev, Tatiana Kazakova, Olga Marshinskaia

**Affiliations:** Federal Research Center of Biological Systems and Agrotechnologies of the Russian Academy of Sciences, 460000, Orenburg, Russia

**Keywords:** immunity, immunodeficiency, inflammation, poultry

## Abstract

**Background and Aim:**

Modern scientific research focuses on a detailed study of the immune system, the mechanisms of immunosuppression, and the search for an effective means to restore disturbed immune homeostasis in farm animals. The present study examined the cytokine and immunoglobulin (Ig) profiles of healthy broiler chickens during physiological development.

**Materials and Methods:**

Arbor Acres broilers (n = 28) were used in the study to achieve this objective. The immune status of broiler chickens was assessed on 7, 14, 28, and 42 days of age, including serum levels of cytokines, Igs, and lysozyme by enzyme immunoassay.

**Results:**

We observed a decrease in the efficiency of immune system functioning of birds with increasing age. The most pronounced immunological deficiency in the body of broiler chickens is noted at the age of 7–14 days, which is associated with immaturity of the immune system and is characterized by the fact that non-specific humoral and specific cellular defense factors are at a rather low level. The levels of lysozyme, interleukin (IL)-2, IL-10, and IgA in blood serum at this age were minimal; starting from 28 days of age, there is a specific humoral immune deficiency, which is compensated by strengthening of cellular defense factors. The serum level of IgY intensively decreases against the background of an increase of lysozyme, IL-2, IL-4, and IL-10.

**Conclusion:**

During postnatal ontogenesis, the immune system of broiler chickens undergoes dynamic changes that have an age direction and phase character. Changes in the immune system may affect immunocompetence, disease susceptibility, and, consequently, productivity.

## Introduction

Industrial poultry farming is developing intensively, providing high-quality products to the population in increasingly shorter periods of time [1, 2]. However, under conditions of increasing productivity as well as the impact of various environmental stress factors (not ideal temperature and humidity conditions in the premises, change of diet and feeding level, technological methods), there is a decrease in the adaptive capabilities of the body, depletion of its functional reserves, which affects the health status of poultry [3–5], with great economic implications [6].

First, secondary (acquired) immunodeficiencies have been noted in birds, which are characterized by a decrease in immune system function and resistance to various infections [7, 8]. The main task of the immune system is to recognize and eliminate foreign substances of antigenic nature [9]. This function is performed, on the one hand, with the help of innate immunity and, on the other hand, adaptive (acquired). The normal functioning of the immune system is possible only if all links between specific immune reactions and non-specific immune reactivity factors are interconnected [10]. However, in recent years, scientists have begun to note that high-yielding hybrids are characterized by lower resources for adaptation (resource allocation theory), and cases of weak immune response to vaccinations are more often recorded [11–13].

The widespread prevalence of immunodeficiency challenges researchers to develop a sound and evidence-based methodology for the early detection and timely correction of immune system deficiencies. Modern scientific research in this area is primarily aimed at the detailed study of the immune system of farm animals, mechanisms of immunosuppression, and search for effective means of restoration of disturbed immune homeostasis [14–16]. Despite progress over the past few decades, numerous key questions regarding cell-mediated and humoral immunity in birds need to be addressed. A better understanding of the functioning of the avian immune system will facilitate the identification of causal factors in the development of immunodeficiency, as well as the development of effective strategies to combat immunopathological conditions.

Therefore, this study aimed to investigate the cytokine and immunoglobulin (Ig) profiles of healthy broiler chickens during ontogenesis.

## Materials and Methods

### Ethical approval

The experimental studies were conducted in accordance with the instructions and recommendations of the Russian regulations (Order of the Ministry of Health of the USSR No.755 of August 12, 1977, “On measures to further improve the organizational forms of work using experimental animals”), the protocols of the Geneva Convention, and the principles of good laboratory practice (National Standard of the Russian Federation GOST R 53434-2009). All animal procedures were performed in accordance with the rules of the Animal Ethics Committee of the FSSI FRC BST RAS.

### Study period and location

This study was conducted in June 2023. The studies were conducted in the laboratory of biological testing and expertize of the Federal State Budgetary Scientific Institution “Federal Scientific Center for Biological Systems and Agrotechnologies of the Russian Academy of Sciences” (accreditation certificate of the State Standard of Russia - PA.RU2159 dated December 02, 2015).

### Experimental animals

This study was conducted on 28 broilers of the Arbor Acres cross (CJSC “Poultry Farm Orenburgskaya”) in the Russian Federation. The study design included four groups (n = 7) of broiler chickens of different ages (7, 14, 28, and 42 days). Standard management procedures were used throughout the experiment. Water was supplied through nipple drinkers. Water and feed were provided *ad libitum*. Broiler chickens were reared on a three-phase diet throughout the experimental period according to the nutritional recommendations. A starter diet, a grower diet, and a finishing diet were provided from days 0–10, 11–20 to 21–35. The feed composition included wheat, barley, corn, soybeans, soybean meal, sunflower meal, sunflower oil, limestone flour, table salt, meat meal, amino acids, vitamins, and mineral premix (Table-1).

**Table 1:** Composition of the diet.

Component	Starter feed	Grower feed	Finisher feed
NE, kcal/100 g	305	307	311
Crude protein, %	23.0	21.7	18.0
Crude fat, %	5.15	5.99	6.48
Crude fiber, %	3.55	3.73	4.77
DM, %	89.29	89.29	91.57
Lysine, %	1.43	1.32	1.08
Methionine + cysteine, %	1.08	1.01	0.86
Threonine, %	0.98	0.9	0.77
Ca, %	1.0	0.91	0.91
P, %	0.83	0.82	0.74
K, %	0.76	0.71	0.6
Na, %	0.17	0.2	0.15
Biologically active substances			
Vitamin A, IU/kg	14,400	12,000	12,000
Vitamin D3, IU/kg	4,800	4,000	4,000
Vitamin E, mg/kg	72.0	60.0	60.0
Vitamin K3, mg/kg	2.4	2.0	2.0
Vitamin B1, mg/kg	2.4	2.0	2.0
Vitamin B2, mg/kg	9.6	8.0	8.0
Vitamin B3, mg/kg	36.0	30.0	30.0
Vitamin B4, mg/kg	600.0	500.0	500.0
Vitamin B5, mg/kg	12.0	10.0	10.0
Vitamin B6, mg/kg	3.6	3.0	3.0
Vitamin B12, mg/kg	0.03	0.025	0.025
Vitamin B9, mg/kg	0.6	0.5	0.5
Vitamin H, mg/kg	0.12	0.1	0.1
Fe, mg/kg	30.0	25.0	25.0
Cu, mg/kg	12.0	10.0	10.0
Zn, mg/kg	96.0	80.0	80.0
Mn, mg/kg	96.0	0	80.0
Co, mg/kg	1.2	0	1.0
I, mg/kg	0.84	0	0.7

DM=Dry matter, NE=Net energy

Blood was collected from the subclavian vein at selected ages using tubes with clotting activator and Vacuette gel (Greiner Bio-One International AG, Austria).

The immune status of birds was assessed, including determination of serum levels of cytokines (interleukin [IL]-2, IL-4, IL-10, interferon-gamma [IFN-γ]), Igs (IgA, IgY) and lysozyme by enzyme-linked immunosorbent assay with the help of tablet spectrophotometer INNO (LTek, Republic of Korea) using the appropriate reagent kits enzyme-linked immunosorbent assay (ELISA) kit, Chicken IL-2 (Cloud-Clone Corp., USA), ELISA kit, Chicken IL-4 (Cloud-Clone Corp.), ELISA kit, Chicken IFN-γ (Cloud-Clone Corp.), ELISA kit, Chicken IL-10 (Cloud-Clone Corp.), ELISA kit, Chicken IgY (BlueGene Biotech, China), ELISA kit, Chicken IgA, (BlueGene Biotech), respectively. Temperature and relative humidity corresponded to the norms recommended for broilers. The photoperiod program complied with European Social Security Regulation 43/2007 (Council Directive 2007/43/EU laying down the minimum rules for the protection of chickens kept for meat production).

### Statistical analysis

Data were analyzed using Statistica version 10 (StatSoft Inc., USA). The Shapiro–Wilk test was used to check the normality of the obtained data. The hypothesis that the data belonged to a normal distribution was rejected in all cases with a probability of 95%, which justified the use of non-parametric procedures for downstream statistical analyses; therefore, we examined differences among group means using Mann–Whitney U-tests. Data are presented as median and 25^th^–75^th^ centiles (Q_25_-Q_75_).

## Results

**Figure-1:**
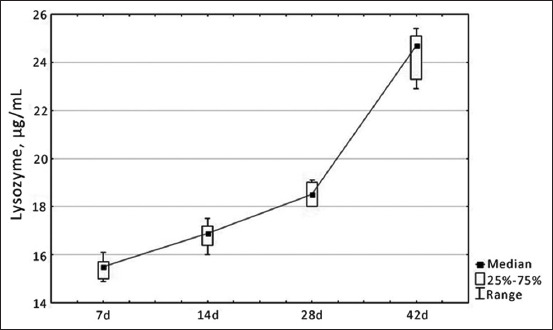
Dynamics of changes in the level of lysozyme in the blood serum of healthy broiler chickens depending on age, µg/mL.

The development of the immune system of broiler chickens was quite stable, as confirmed by the parameters of both non-specific cellular and humoral immunity and the indicators of specific immune defense. The increase in lysozyme levels, which is a parameter of specific humoral immunity, was gradual, reaching maximum values on 42 days of age (24.7 μg/mL) in broiler chickens (Figure-1).

**Figure-2:**
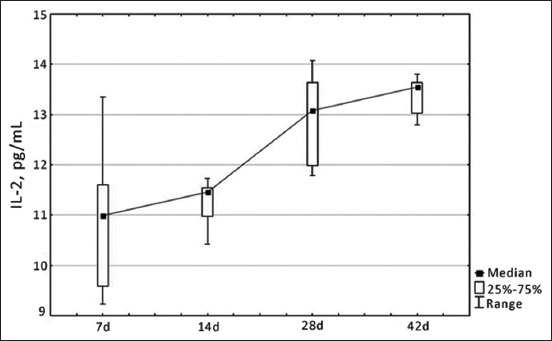
Dynamics of changes in interleukin-2 level in blood serum of healthy broiler chickens depending on age, pg/mL.

**Figure-3:**
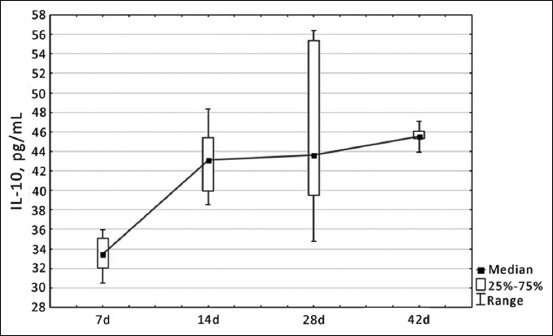
Dynamics of changes in interleukin-10 level in blood serum of healthy broiler chickens depending on age, pg/mL.

Similar dynamics were observed among the indicators of specific cellular immunity: the increase in IL-2 and IL-10 content was also gradual, reaching a maximum value of 13.55 pg/mL and 45.52 pg/mL by the 42^nd^ day of development, respectively (Figures-2 and 3). It should be noted that the lowest values of these indicators were established from 7 to 14 days of physiological development of chickens, which is justified by the immaturity of the immune system of birds and the inability to produce a sufficient amount of cytokines.

**Figure-4:**
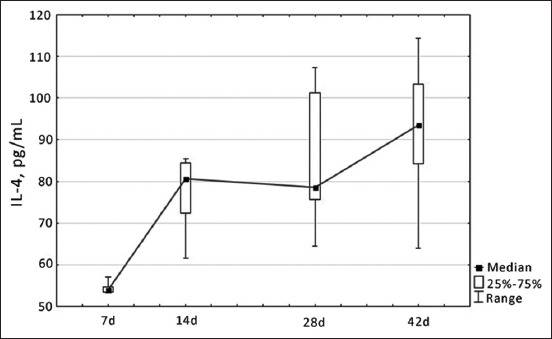
Dynamics of changes in interleukin-4 level in blood serum of healthy broiler chickens depending on age, pg/mL.

As indicated, IL-4 was increased up to 14 days of age in broiler chickens. However, when considering the dynamics of IL-4 content further in the blood serum of birds, unstable changes were revealed – the increase in serum IL-4 content on the 14^th^ day of physiological development of broilers alternated with a decrease in this indicator by the 28^th^ day of development, which, in turn, was replaced by a persistent increase in this indicator by the 42^nd^ day of development (Figure-4).

**Figure-5:**
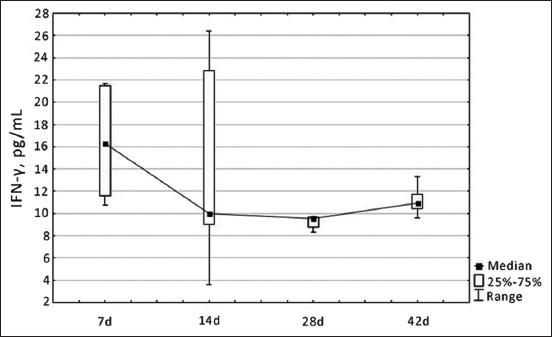
Dynamics of changes in the level of interferon-gamma in blood serum healthy broiler chickens depending on age, pg/mL.

Figure-5 clearly shows a completely different trend in the dynamics of IFN-γ, which gradually decreased during the ontogenetic development of birds and reached a minimum value by the 28^th^ year of life (9.52 μg/mL). However, at the age of 42, the content of this indicator was again increased.

Determining Ig levels is one of the main and reliable methods of assessing the B-system of immunity for diagnosing all forms of immunodeficiency.

**Figure-6:**
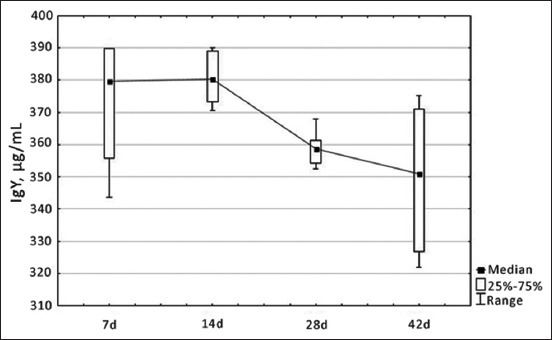
Dynamics of changes in immunoglobulin Y level in blood serum of healthy broiler chickens depending on age, µg/mL.

Despite the high level of IgY on 7 days of age, broiler chickens experienced a depletion of protective forces in the first 2 weeks of life due to the breakdown of ovarian globulins and morphofunctional immaturity of the immune system, resulting in a gradual decrease in the Ig content, which reached minimum values by the 42^nd^ day of physiological development (Figure-6).

**Figure-7:**
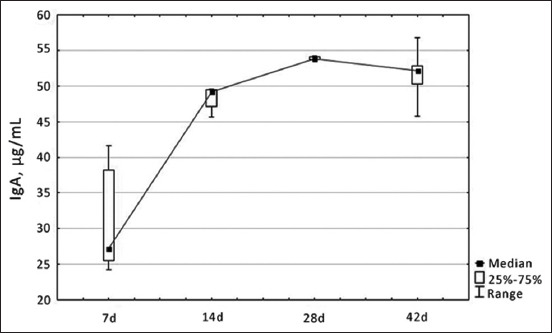
Dynamics of changes in immunoglobulin A level in blood serum of healthy broiler chickens depending on age, µg/mL.

The serum IgA level gradually increased during the ontogenesis of chickens but decreased starting from 28 days of physiological development (Figure-7).

## Discussion

During postnatal ontogenesis, the immune system of broiler chickens undergoes dynamic changes that have an age direction and phase. The most pronounced immunological deficiency in the body of broiler chickens is noted at the age of 7–14 days, which is associated with immaturity of the immune system and is characterized by a fairly low level of non-specific humoral, specific cellular, and humoral defense factors. This suggests that the immune function in broiler chickens did not develop well during this period because the immune system has not yet matured, and the chick cannot produce sufficient lysozyme, cytokines, and Igs. Low levels of these parameters may slow down the differentiation of stem cells into mature immune cells and lead to poor disease resistance in broilers. Starting on 28 days of age, there is a specific humoral immune deficiency, which is compensated by the strengthening of cellular defense factors. The serum IgY level decreases intensively against the background of increased levels of lysozyme, IL-2, IL-4, and IL-10.

Non-specific (innate) immunity is the most ancient branch of the immune system and is phylogenetically associated with primitive manifestations of protozoan organisms’ life activity. Over time, non-specific (innate) immunity has become more complex and forms a variety of effective defense mechanisms. This immune system link is considered the body’s front line of defense, represented by numerous factors and systems, including lysozyme [17]. Lysozyme, also known as muramidase or 1,4-β-N-acetylmuramidase, serves as one of the key humoral factors of innate immunity and performs important biological functions in the body (bactericidal properties, stimulating effect on phagocytosis, neutralization of some microbial toxins, and anti-inflammatory effect) [18].

Specific (acquired) immunity is represented by cellular immunity due to T-lymphocytes and humoral immunity due to B-lymphocytes [19]. Although humoral (antibody-mediated) immunity is important in the body’s defense against many bacterial and viral infections, many (especially intracellular infections) involve primarily cellular immunity, which provides the body’s resistance to the action of infectious agents and in recovery [20]. Experimental studies have shown that the body responds to the introduction of an infectious agent with a universal, genetically programmed reaction in the form of inflammation [21]. The cytokine system plays a leading role in the maintenance of homeostasis [22]. Cytokines are the main signaling molecules of the immune system, modulating the activity of its cells, determining the activation of the innate immune response, and the development of adaptive immune response [23]. Types of immune response are associated with the preferential participation of helper T-lymphocyte clones of type 1 (Th1) or type 2 (Th2), which differ in the cytokine produced and their role in stimulating the development of cellular or humoral immune response [24]. Th1 activation leads to the development of a cellular response, whereas Th2 synthesis mainly stimulates the humoral link of immunity [25]. IL-2 is a Th1-type cytokine that participates in all inflammatory reactions and mainly stimulates the cellular link of specific immunity [26]. IL-10 inhibits cellular immunity suppresses the production of proinflammatory cytokines, prevents the differentiation of monocytes into tissue macrophages and apoptosis, and enhances the production of IL-2 and IFN-γ [27]. IL-4 is a Th2 cytokine that regulates allergic type of inflammation and mainly stimulates the humoral link of specific immunity. These immune functions are evolutionarily designed to rapidly develop an inflammatory response. IL-4 stimulates antigen-activated B-lymphocyte proliferation and induces Ig synthesis [20, 28]. IFN-γ is a type II IFN that regulates specific immune response and non-specific resistance. It stimulates the activity of T- and B-lymphocytes, and IL-4 antagonist maintains Th1/Th2 balance [29, 30].

Serum IgY and IgA are humoral immune molecules produced by active humoral immune cells in the body’s immune organs and tissues and are important indicators of the functional status of the humoral immune system. The most important functions of antibodies are neutralization of toxins and viruses, opsonization of microorganisms to enhance phagocytosis, complement activation, and prevention of adherence of microorganisms to the mucosal surface [31]. IgY is the predominant isotype in avian serum and egg yolk and the major isotype in the secondary immune response, which determines its functional similarity to mammalian IgG [32]. Maternal antibodies play an important role in the protection of chicks against external threats, particularly in the first 3 weeks after hatching. Maternal antibodies decreased gradually with increasing age of chickens. IgA plays a crucial role in protecting the mucosal surface from toxins, viruses, and bacteria by directly neutralizing or preventing binding to the mucosal surface [33, 34].

Changes in the immune system may affect immunocompetence, disease susceptibility, and, consequently, productivity. To ensure optimal productivity, it is important to monitor and, if necessary, modulate the immune response to maintain homeostasis. Thus, promoting the rapid development and maturation of the immune system of chickens after hatching is very important for their health at an early age. Because broilers have a short feeding period, the innate immune function is very important for the formation of disease resistance. Nutritional interventions can effectively promote the development and maturation of the early immune system of broiler chickens and, as soon as possible, increase the innate immunity of the chickens.

## Conclusion

The results of the present study indicate that during postnatal ontogenesis, the immune system of broiler chickens undergoes dynamic changes with age and phase characteristics. Changes in the immune system may affect immunocompetence, disease susceptibility, and, consequently, meat productivity.

It is important to determine the normal concentrations of cytokines and Igs in the blood serum of poultry at different ages to assess changes in their levels during a particular pathological process. This information is equally important for understanding normal immunological changes at different ages and the process of immunological maturation. Analysis of dynamic immune status indicators is always more informative in diagnostic and prognostic terms. The established regularities of changes in the cytokine and Ig profiles of broiler chickens in the course of physiological development complement and generalize the theory of individual development of the bird body. Thus, the data obtained may contribute to veterinary medicine as well as to the biology of individual development of farm birds, in particular, Arbor Acres broiler chickens.

## Data Availability

The authors can confirm that all relevant data are included in the article.

## Authors’ Contributions

All authors contributed to the study’s conception and design. SL: Supervised the study and edited the manuscript. TK: Data and sample collection, laboratory tests, data analysis and interpretation, critical review, and manuscript drafting. OM: Conceptualization, laboratory tests, data analysis and interpretation, manuscript drafting, editing, and revision. All authors have read, reviewed, and approved the final manuscript.
